# Abdominal Tuberculosis With a Cocoon

**Published:** 2012-08-30

**Authors:** Arif Hussain Sarmast, Hakim Irfan Showkat, Afaq Sherwani, Mohammad Yousuf Kachroo, Fazl Q Parray

**Affiliations:** 1 Department of Surgery, SKIMS Soura, Soura, India

**Keywords:** Abdomen, Tuberculosis, Laparotomy

Dear Editor,

Abdominal cocoon or sclerosing encapsulating peritonitis is a rare condition of unknown cause in which intestinal obstruction results from the encasement of variable lengths of bowel by a dense fibrocollagenous membrane that gives the appearance of a cocoon. This condition is not often suspected preoperatively, and therefore the diagnosis is usually made at laparotomy [1]. The abdominal cocoon was first described by Owtschinnikow in 1907 as “peritonitis chronica fibrosa incapsulata” [2].

A 30 year old male presented with pain left lower abdomen and vomiting of 3 days duration. He had similar episodes of pain and vomiting in the past that were managed conservatively, with a diagnosis of subacute intestinal obstruction (SAIO). On examination he was thin built and looked undernourished. His vitals were normal and he had mild pallor with Hb of 10.9 with no lymphadenopathy. Abdomen was distended, with a sausage shaped intra-abdominal lump felt in left umbilical region, size 6x4cm, with concavity towards midline. Margins of the lump were ill defined, soft in consistency, non-pulsatile, mobile. Bowel sounds were not present and there was no shifting dullness. Hernial sites and per rectal examination was normal. The patient had no previous history of hepatic disease, abdominal surgery, peritoneal dialysis, ventriculoperitoneal and peritoneovenous shunting. Additionally, no clinical features of connective tissue disease could be identified. A family history of chest tuberculosis was, however, positive. Plain radiograph abdomen showed multiple air and fluid levels in the erect position. Chest radiographs were however normal without any evidence of pulmonary tuberculosis. Ultrasound abdomen showed gas filled loops of small bowel, suggestive of small bowel loop obstruction. A CECT (contrast enhanced computed tomography) abdomen showed gut wall thickening (Figure 1)

**Figure 1:**
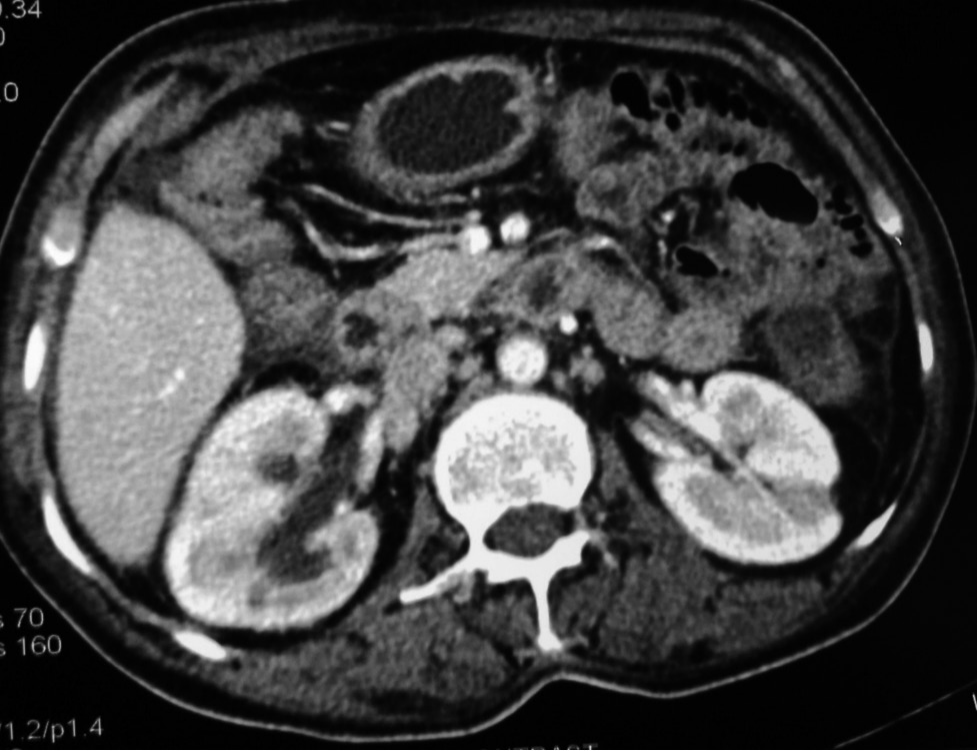
CECT abdomen showing thickened gut wall

Diagnosis of acute intestinal obstruction was made. Exploratory laparotomy was done. The whole of small bowel was adhered together like a cocoon (Figure 2 and 3) from the duodeno-jejunal flexure to the ileo-caecal region, encapsulated within a peritoneal membrane with adhesions which were broken and the thick membrane was resected. Mesenteric Lymphadenopathy was noted. Histology of the membrane revealed caseating granuloma and giant cells with mild fibrosis and nodal microscopy revealed same features. A diagnosis of abdominal cocoon secondary to abdominal tuberculosis was suspected. Postoperatively, the patient was initiated on anti-tuberculous treatment for 9 months. For initial phase of 2 months, Isoniazid (H) [600mg], Rifampin (R) [600mg], Pyrazinamide (Z) [2000mg], and Ethambutol (E) [1600mg] were used thrice a week and in continuation phase of 7 months only Isoniazid (H) [600mg] and Rifampin (R) [600mg] were used thrice a week. During 3 year of postoperative follow-up period, the patient remained asymptomatic.

**Figure 2:**
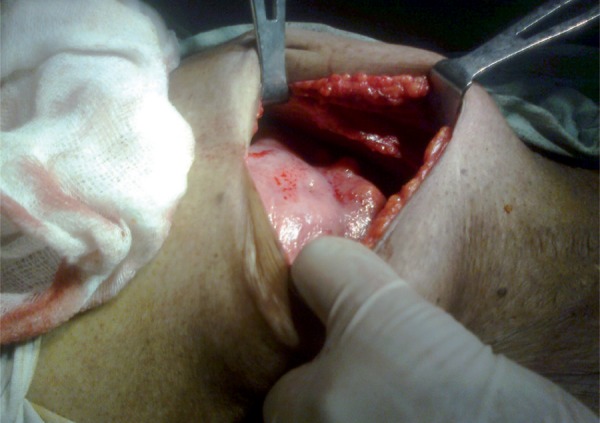
Abdominal cocoon seen on laparotomy

**Figure 3:**
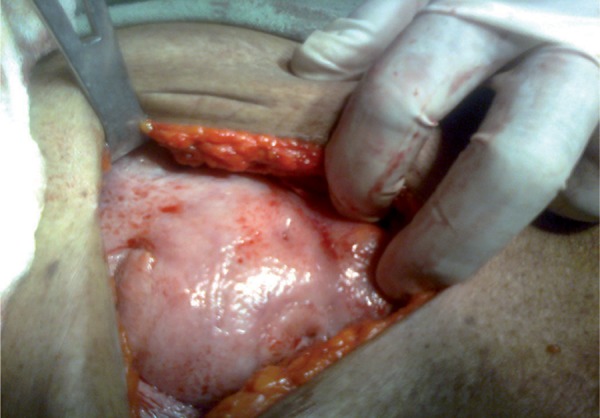
Whole of small gut seen as a cocoon

Abdominal cocoon is a rare disease, characterized by a thick fibrotic membrane that wraps the small bowel in a concertina-like fashion. Terms such as sclerosing peritonitis [3], encapsulating peritonitis [4] and sclerosing encapsulating peritonitis [5] have also been used to describe this condition. Yip and Lee listed four main clinical features that help identify abdominal cocoon preoperatively. These features are its occurrence in a relatively young girl without an obvious cause of intestinal obstruction, a history of similar episodes that resolved spontaneously, a presentation with abdominal pain and vomiting but rarely the four cardinal symptoms of intestinal obstruction, and the presence of a nontender soft mass on abdominal palpation [6]. The ability of CT to depict the cause of a small-bowel obstruction, with a sensitivity of 73–95% for high-grade small-bowel obstruction [7] makes it an important diagnostic tool. The condition has been classified as primary and secondary based on whether it is idiopathic or has a definite cause. Primary or idiopathic cocoon occurs in young girls, especially those from tropical and subtropical areas, presenting with small-bowel obstruction and a palpable abdominal mass without any obvious cause. Because of the peculiar age and sex distribution of the disease, it was postulated that the condition is due to retrograde menstruation with subclinical, viral peritonitis resulting in the development of an encapsulating membrane on the intestine [8]. Secondary abdominal cocoon may occur as a serious complication of CAPD [9]. The prevalence of abdominal cocoon in patients undergoing CAPD ranges from 0.5% to 2.8% [10][11]. Conservative management of abdominal cocoon often fails. Surgery includes freeing the bowel from the thick encasing membrane and the release of the obstruction. Finger dissection is done with minimal blood loss. The bowel serosa is not injured at any stage. Extensive surgery and unnecessary bowel resection are associated with a high incidence of anastomotic failure and should be avoided [12].

## References

[1] Hur J, Kim KW, Park MS, Yu JS (2004). Abdominal cocoon: preoperative diagnostic clues from radiologic imaging with pathologic correlation. AJR Am J Roentgenol.

[2] Lalloo S, Krishna D, Maharajh J (2002). Case report: abdominal cocoon associated with tuberculous pelvic inflammatory disease. Br J Radiol.

[3] Eltringham WK, Espiner HJ, Windsor CW, Griffiths DA, Davies JD, Baddeley H (1977). Sclerosing peritonitis due to practolol: a report on 9 cases and their surgical management. Br J Surg.

[4] Verger C, Celicout B (1985). Peritoneal permeability and encapsulating peritonitis. Lancet.

[5] Holland P (1990). Sclerosing encapsulating peritonitis in chronic ambulatory peritoneal dialysis. Clin Radiol.

[6] Yip FW, Lee SH (1992). The abdominal cocoon. Aust N Z J Surg.

[7] Burkill GJ, Bell JR, Healy JC (2001). The utility of computed tomography in acute small bowel obstruction. Clin Radiol.

[8] Ahmed MN, Kaur S, Zargar HU (1984). Abdominal cocoon: an unusual intestinal obstruction (a case report).. J Postgrad Med.

[9] Hamaloglu E, Altun H, Ozdemir A, Ozenc A (2002). The abdominal cocoon: a case report. Dig Surg.

[10] Kawanishi H, Kawaguchi Y, Fukui H, Hara S, Imada A, Kubo H (2004). Encapsulating peritoneal sclerosis in Japan: a prospective, controlled, multicenter study. Am J Kidney Dis.

[11] Chin AI, Yeun JY (2006). Encapsulating peritoneal sclerosis: an unpredictable and devastating complication of peritoneal dialysis. Am J Kidney Dis.

[12] Chopra D, Lakhe J, Sharma S, Salgotra K, Raju M (2006). Abdominal Cocoon. Med J Armed Forc India.

